# The Epidemiology of Skateboarding Injuries: A 10-Year Review at a Major Australian Centre

**DOI:** 10.7759/cureus.55624

**Published:** 2024-03-06

**Authors:** Jonathan Quinn, Michael Singh, Jake Bennetto, Elliot Duong, Francios Tudor, Simon Platt

**Affiliations:** 1 Orthopaedics, Gold Coast University Hospital, Gold Coast, AUS; 2 Orthopaedics, Queen Elizabeth II Jubilee Hospital, Brisbane, AUS

**Keywords:** sports, trauma, fracture, epidemiology, musculoskeletal injuries, skateboarding

## Abstract

Aims

The purpose of this study was to attempt to quantify the impact of skateboarding-related injuries on our local orthopaedic service.

Method

Every presentation to the emergency departments of Gold Coast health public hospitals was retrospectively reviewed to determine whether these were skateboarding-related injuries. Between 2008 and 2018, 5,026 injuries were identified. Patient demographics, mechanism of injury, anatomic location of the injury, treatment, and medical specialty providing treatment were collected and analysed.

Results

Our investigation demonstrated that skateboarding injuries are common and cause significant trauma in a large percentage of cases. These injuries place a high demand on orthopaedic and emergency departments. This cohort demonstrated a male-to-female ratio of over 4:1. The most common injury type was fracture (44%), of which the upper limb (56%) was the most commonly affected anatomical region. Injuries to the wrist and hand account for 57% of all upper limb injuries, and ankle injuries account for 45% of lower limb injuries. Traumatic brain injuries and concussions account for 62% of all head injuries in this cohort. The mean age was 19 years, with a predominance of 18-25 years old. Only 14% of injuries occurred in those 30 and older. Definitive orthopaedic care, involving inpatient admissions and/or outpatient clinic follow-up, was required in at least 48% of cases, whilst 50% were able to be treated in the emergency department. Additionally, 86% of head injuries were able to be managed in the emergency department, and 11% required specialist neurosurgical management.

Conclusion

Skateboard-related injuries represent a significant burden of trauma to the local community, predominantly musculoskeletal injuries within the adolescent and young male adult population. This research may direct future strategies for harm minimization, specifically targeted at this demographic of patients.

## Introduction

Skateboard-related injuries are a common cause for presentation to the emergency departments in the Gold Coast Hospital and Health Service, Australia. Factors contributing to this include the growing population, community support of skateboarding activities, and questionable use of appropriate safety equipment.

The Gold Coast is one of Australia’s fastest growing cities, with a population of 650,000, and a growth of 2% per annum [1]. The Gold Coast Hospital and Health Service (GCHHS) provides healthcare to this population, with over 170,000 patient presentations to the emergency departments in 2017-2018, thereby making it one of the busiest in Australia [2].

Skateboarding was popularised by surfers in the mid-20th century as a means to ‘surf’ when the waves were flat [3]. Given the geographic location and prominent surf culture, it is no surprise that surfing, and therefore skateboarding, are both quintessential Gold Coast pastimes.

We aim to retrospectively investigate and quantify skateboard-related injuries and presentations to Gold Coast public hospitals. This information will assist in understanding the epidemiology of these injuries and the associated cost, which will inform future injury mitigation strategies.

## Materials and methods

This study is a retrospective chart review of patients presenting to the emergency departments of all hospitals in the Gold Coast University Hospital and Robina Hospital (GCHHS), Australia, from 1 January 2008 to 31 December 2018. We identified patients for this study from the Emergency Department Information Systems (EDIS) triage. This system is used by triage nurses to evaluate patients upon presentation and triage them according to the urgency of required care. Based upon these records, all patients are recorded onto the database for the hospital. Data were extracted from this system using a data search in the ‘Presenting Complaint/Problem’ section for the terms: ‘SKATE’, ‘SKATEBOARDING’, ‘SKATEPARK’ or ‘SKATER’.

A total of 5,742 patient presentations were then identified as relating to these terms. These presentations were retrospectively screened using an electronic medical record (EMR) to assess whether they were suitable for inclusion in the analysis.

Inclusion criteria were all patient presentations to the emergency department for an injury sustained during skateboard use. Exclusion criteria were cases not related to skateboard use, inadequate documentation of injury or mechanism contained in EMR, or repeat presentations for a previously documented injury. Of 5,742 identified patient presentations, 716 were excluded through these criteria, resulting in 5,026 skateboard-related injury presentations.

Data were then extracted from the EMR by a team of reviewers. Included cases underwent thorough EMR chart review to document patient demographics, presenting complaint, injury type, anatomical location of injury, treatment, and treating team providing care. This included a subset comparison to ensure homogeneity and accuracy of data extraction.

Ethics approval was granted prior to study commencement by the institutional ethics committee (Gold Coast Hospital and Health Service Human Research Ethics Committee (EC00160), approval LNR/2018/QGC/45215).

## Results

**Figure 1:**
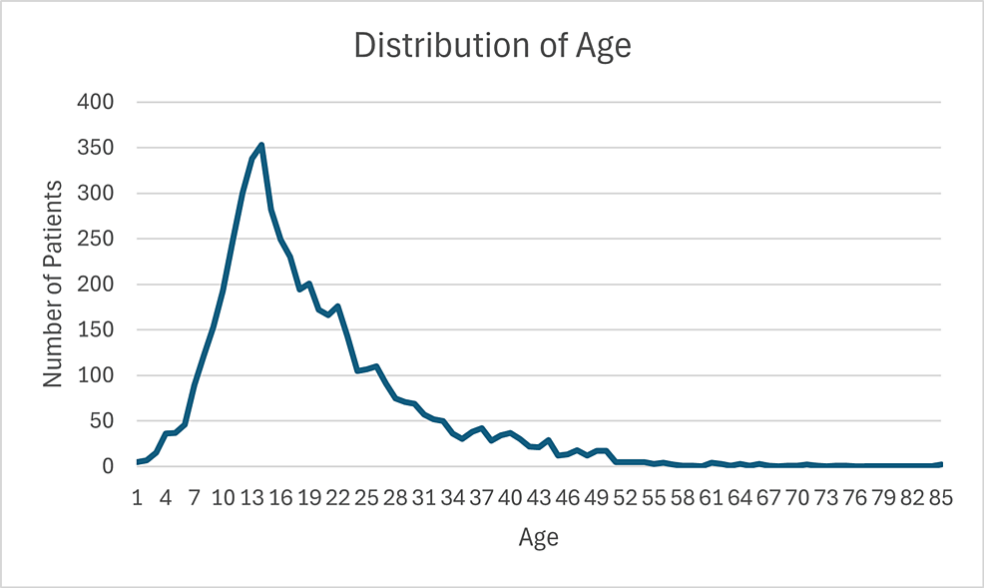
Distribution of age Age distribution of skateboarding-related injuries

Demographical data are presented in Table 1. There were a total of 5,026 skateboarding-related injuries identified. Of these, 4,047 (80.5%) were men, and 979 (9.5%) were women, with a ratio of over 4:1. The mode age was 14 years, with a mean age of 19.4 years and a range from one to 85 years of age. The distribution of these ages is presented in Figure 1. Patients over the age of 30 years contributed 14% of our cohort.

**Table 1:** Patient demographics

Characteristics	Patients
Total cases, n	5026
Age (years), mean (median)	19.4 (17)
Sex (male), n(%)	4047 (80.5)
Sex (female), n(%)	979 (9.5)

Injury type

**Figure 2:**
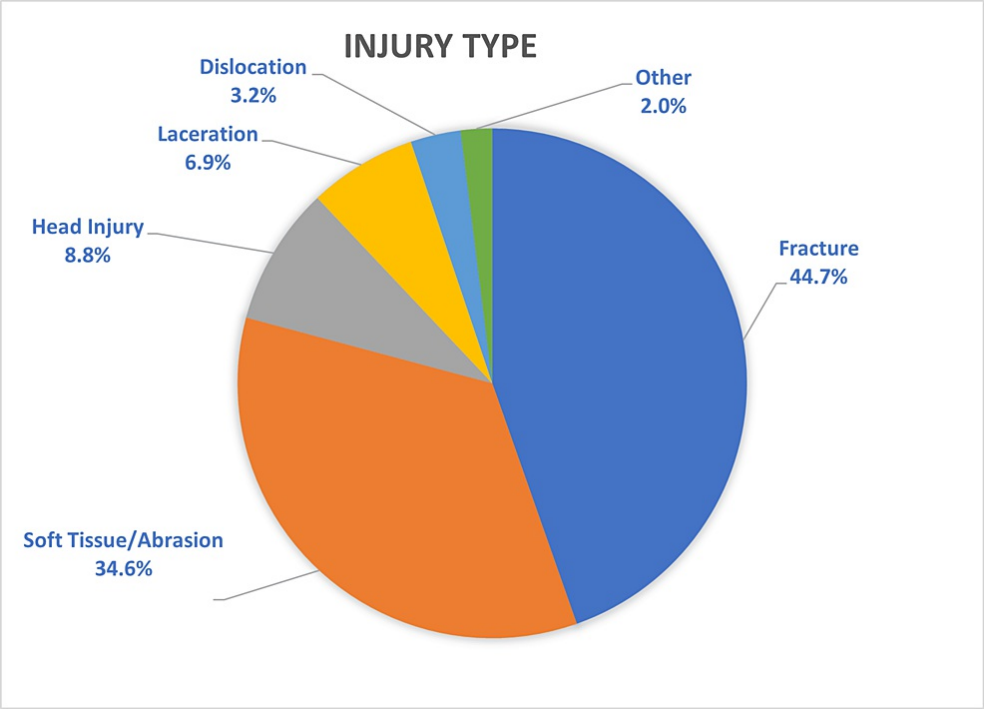
Distribution of injury types

**Figure 3:**
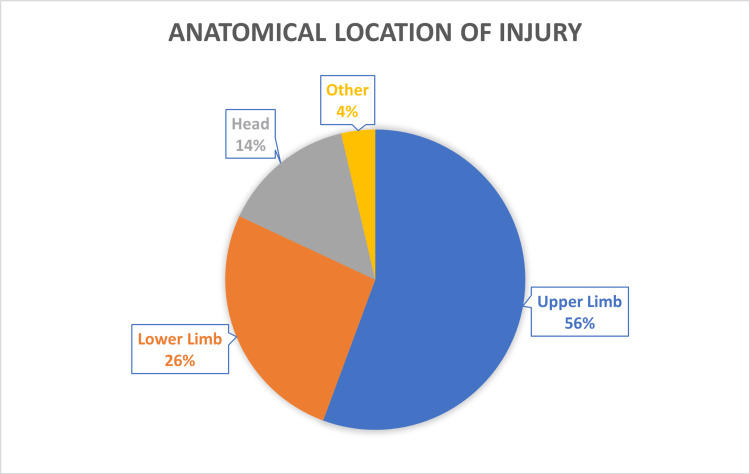
Anatomical location of injury

**Figure 4:**
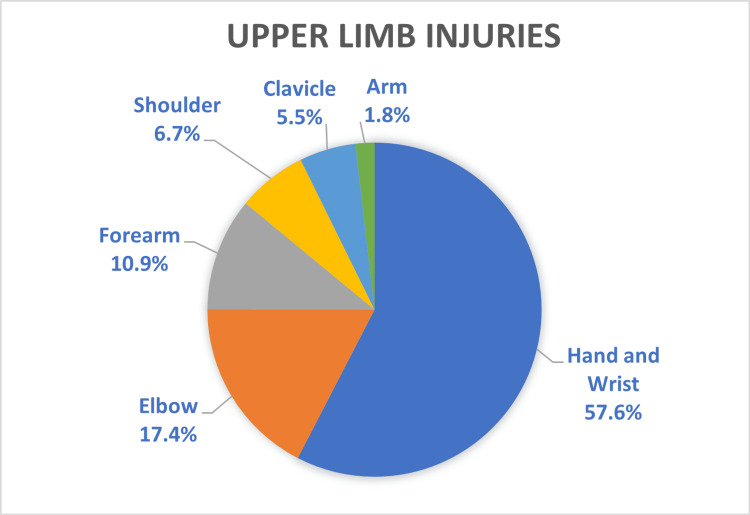
Percentage distribution of upper limb injuries from skateboard-related injuries

**Figure 5:**
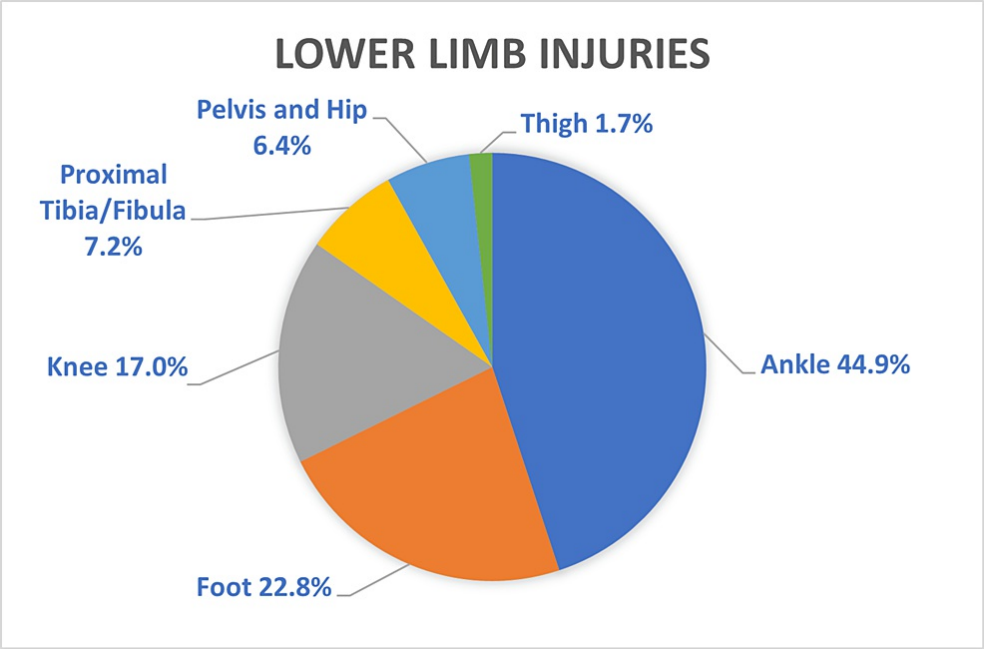
Percentage distribution of lower limb injuries from skateboard-related injuries

**Figure 6:**
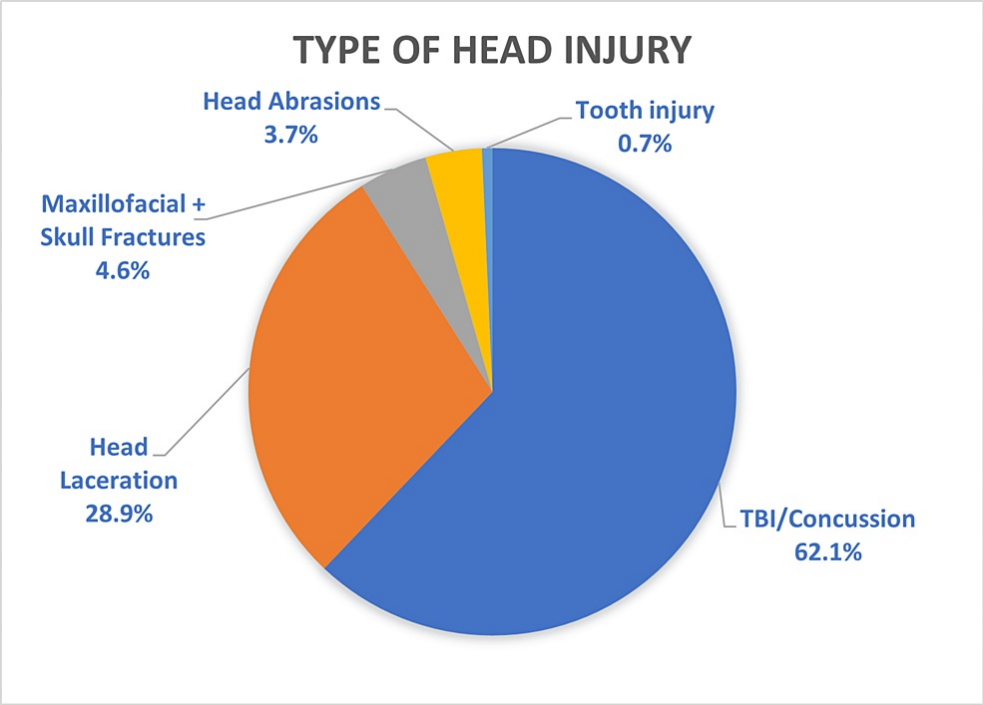
Type of head injury

Injury data are presented in Table 2. Fractures (n=2,241) were the most common type of injury, followed by soft tissue injuries (n=1,735) and head injuries (n=442), which were inclusive of all types of injuries to the head (Figure 2). The most common anatomical region of the body affected was the upper limb, comprising over half the total number of presentations (56%, n=2,797) (Figure 3). Of those presentations involving the upper limb, hand and wrist injuries constituted nearly two-thirds of all upper limb injuries (n=1,611) (Figure 4). The most common location of lower limb injury was the ankle (44%, n=594), followed by the feet (22%, n=301) (Figure 5). Head injury breakdown showed that the majority of head injuries were traumatic brain injuries/concussions (62.1%, n=449) (Figure 6).

**Table 2:** Injury data

Injury type	Total cases (n)
Fracture	2241
Soft tissue injuries/abrasions	1735
Head injury	442
Laceration	345
Dislocation	160
Other	100
Anatomical location	Total cases (n)
Upper limb	2797
Lower limb	1322
Head	723
Other	184
Upper limb injury location	Total cases (n)
Hand and wrist	1611
Elbow	488
Forearm	306
Shoulder	188
Clavicle	153
Arm	51

Lower limb injury location	Total cases (n)
Ankle	594
Foot	301
Knee	225
Proximal tibial/fibula	95
Pelvis + hip	84
Thigh	23

Head injury type	Total cases (n)
Concussion/traumatic brain injury	449
Laceration	209
Maxillofacial/skull fracture	33
Head abrasions	27
Tooth injuries	5

Treating team

Additionally, 48% (n=2,408) of patients were managed by the orthopaedic department, while 50% (n=2,508) remained under the care of the Emergency Department. Approximately 1% (n=48) of patients were managed by the neurosurgical/intensive care teams. In regard to traumatic brain injuries/concussions, 87% (n=390) were managed in the emergency department, and 11% (n=49) were managed by neurosurgery.

## Discussion

This study is the largest single-centre cohort reporting skateboarding injuries within the literature, with other institution-based cohorts having fewer than 1,000 patients. A series of 80 cases published from Westmead Hospital, NSW, Australia, in 1990 also described fractures of the upper limb being the most common injury pattern and called for harm minimisation strategies to target the at-risk demographics - adolescents and children [4]. Another study in Sweden reviewed a series of 139 cases that described subluxations and dislocations as the most common injuries in their cohort [5]. Larger studies within the literature are based on national electronic databases, with subsequent inherent limitations [6]. Our results also reflect that upper limb injuries are the most common injury pattern, as well as fractures being the predominant injury type. This likely reflects the nature of skateboarding where there is minimal stability of the upper limb while performing skateboarding activities, leading to an increased chance of falling with outstretched upper limbs to brace for falling. The large proportion of fractures found in our study results likely reflects the force involved with accidents and potentially the surface on which the participant is falling. However, no data were gathered on the surface where the accident took place. Further research may be warranted to investigate this.

Assessment of such a vast cohort of patient presentations for skateboarding injuries has allowed for an accurate description of both patient demographics and injury characteristics. While this study was conducted in a specific geographical location, the number of case presentations allows for the results to be applicable to other communities and should be considered to develop a better understanding of the impact of skateboarding and associated risks.

Adolescents and children have previously been identified as a high-risk population for skateboarding-related injuries, with our cohort supporting this population as the main contributor to skateboarding injuries [4-9]. This likely reflects this demographic being the most common age group for skateboarding-related activities.

While we have found upper limb injuries as the most common anatomical location of injury, there is a significant contribution of lower limb injuries as well. The majority of both the upper limb and lower limb injuries were fractures, likely representing the significant force suffered during these injuries. Head injuries, while only accounting for a small amount of presentations, can require substantial initial and ongoing treatment. We found the majority of the head injuries were traumatic brain injuries or concussions. Additionally, 87% of these were managed in the emergency department, with 11% being managed by neurosurgery. This likely reflects that the majority of these head injuries are minor; however, one in 10 injuries are moderate-severe in nature and require specialist management. These injuries can also wreak havoc on patients' rehabilitation and lead to ongoing disability in patients, with potential future loss of income. These injuries have been investigated in a professional athlete cohort calling for the use of protective equipment and the use of surveys to improve preventative measures [10,11].

Of note, our cohort demonstrated a significant contribution of injuries from patients over the age of 30. Injury within this population may result in significant loss of income and impact not only the individual but also the family and community. We would therefore suggest that adults need to consider the suitability of their participation in skateboarding, particularly without protective equipment. Schieber et al. demonstrated that wrist guards and elbows drastically reduced the risk of injury in a similar population [12]. 

The epidemiological findings and subsequent recommendations of Shuman et al. are likely applicable to most populations, and our findings provide support within the Australian community [13].

Limitations

The retrospective nature of this study does incur the associated limitations, most significantly the potential for incomplete data capture. The use of electronic medical records allows for accurate searching of patient data, thereby minimizing the likelihood of missing appropriate presentations. However, given the evolutionary nature of certain injuries, it would be remiss of us to claim that the data are completely error-free. Further, the accuracy of the diagnosis data entered on the EMR is reliant on clinician judgement and therefore cannot be guaranteed. This is mitigated by our large cohort size, thereby limiting the impact of individual cases on our overall trends. While every attempt at the accurate categorisation of data into specific anatomical locations was made, due to the retrospective nature of our study, there may be some minor errors made. Again this is mitigated with that large sample size. There was no data collected on the surface on which the injury occurred or whether the patients were wearing any protective gear.

The nature of the data analysed prevents us from reliably reporting on the mortality rate of skateboard-related injuries. Furthermore, while the data available detail the treating team for the episode of care, it does not detail the involvement of subspecialties providing consultations or other advice. Therefore, it is likely that the involvement of the Orthopaedic Department is under-represented within our results, as well as other teams such as neurosurgery.

Implications for clinical practice

The greatest strength of this study is the number of cases analysed. With such a vast sample size, we have been able to accurately describe the patient and injury characteristics most commonly occurring due to skateboarding. This study enables clinicians and administrators to quantify the impact that skateboarding injuries have on the local orthopaedic service. This research is therefore valuable to the local health service, as well as governing bodies in the development of public safety recommendations and interventions. The large sample size may also make this applicable to other regions. Currently, there is no law in our region requiring skateboarders to wear a helmet or any other protective equipment [14].

The impact of skateboard-related injuries on the individual and the community is significant, with loss of income, loss of contribution to society, and cost of providing healthcare, in the short and long term, as demonstrated in previous studies [15]. These studies also showed that older patients (specifically those aged 26 or older) had the highest associated cost burden, which is relevant to our study given that 14% of patients were over the age of 30 years old. Governance and leadership are necessary within the community to protect not only the health of our citizens but also their ability to earn a living.

Further research should be undertaken prospectively, with a view to quantifying loss of income and other financial parameters as a result of the more common injury patterns. The utility of this would be to enhance the credibility of targeted harm minimisation strategies.

## Conclusions

Our data confirm that the vast majority of skateboard-related injuries affect the musculoskeletal system, with significant requirements for the orthopaedic service. Unsurprisingly, these injuries tend to most frequently befall young males who have landed on an outstretched hand. Approximately one in two skateboard-related injuries in this cohort required input from the orthopaedic team.

From this study alone, enforcing the use of helmets, and encouraging the use of wrist protection braces, could potentially decrease a substantial number of skateboard-related injuries. Further research should be aimed at quantifying which interventions reduce the quantity of skateboarding injuries.
